# Impaired dopamine D_1_ receptor-mediated vasorelaxation of mesenteric arteries in obese Zucker rats

**DOI:** 10.1186/1475-2840-13-50

**Published:** 2014-02-22

**Authors:** Jinjuan Fu, Yu Han, Hongyong Wang, Zhen Wang, Yukai Liu, Xingjian Chen, Yue Cai, Weiwei Guan, Di Yang, Laureano D Asico, Lin Zhou, Pedro A Jose, Chunyu Zeng

**Affiliations:** 1 Department of Cardiology, Daping Hospital, The Third Military Medical University, Chongqing, P.R. China; 2 Chongqing Institute of Cardiology, Chongqing, P.R. China; 3 Division of Nephrology, Department of Medicine, University of Maryland School of Medicine, Baltimore, MD, USA; 4 Department of Physiology, University of Maryland School of Medicine, Baltimore, MD, USA

**Keywords:** Dopamine D_1_ receptor, Vasorelaxation, Hyperinsulinemia, Hyperglycemia, Obesity-related hypertension, Obese Zucker rats

## Abstract

**Background:**

Obesity plays an important role in the pathogenesis of hypertension. Renal dopamine D_1_-like receptor-mediated diuresis and natriuresis are impaired in the obese Zucker rat, an obesity-related hypertensive rat model. The role of arterial D_1_ receptors in the hypertension of obese Zucker rats is not clear.

**Methods:**

Plasma glucose and insulin concentrations and blood pressure were measured. The vasodilatory response of isolated mesenteric arteries was evaluated using a small vessel myograph. The expression and phosphorylation of D_1_ receptors were quantified by co-immunoprecipitation and immunoblotting To determine the effect of hyperinsulinemia and hyperglycemia on the function of the arterial D_1_ receptor, we studied obese Zucker rats (six to eight-weeks old) fed (6 weeks) vehicle or rosiglitazone, an insulin sensitizer (10 mg/kg per day) and lean Zucker rats (eight to ten-weeks old), fed high-fat diet to induce hyperinsulinemia or injected intraperitoneally with streptomycin (STZ) to induce hyperglycemia.

**Results:**

In obese Zucker rats, the vasorelaxant effect of D_1_-like receptors was impaired that could be ascribed to decreased arterial D_1_ receptor expression and increased D_1_ receptor phosphorylation. In these obese rats, rosiglitazone normalized the arterial D_1_ receptor expression and phosphorylation and improved the D_1_-like receptor-mediated vasorelaxation. We also found that D_1_ receptor-dependent vasorelaxation was decreased in lean Zucker rats with hyperinsulinemia or hyperglycemia but the D_1_ receptor dysfunction was greater in the former than in the latter group. The ability of insulin and glucose to decrease D_1_ receptor expression and increase its phosphorylation were confirmed in studies of rat aortic smooth muscle cells.

**Conclusions:**

Both hyperinsulinemia and hyperglycemia caused D_1_ receptor dysfunction by decreasing arterial D_1_ receptor expression and increasing D_1_ receptor phosphorylation. Impaired D_1_ receptor-mediated vasorelaxation is involved in the pathogenesis of obesity-related hypertension.

## Background

There is an increasing incidence of metabolic syndrome (MS) which is characterized by abdominal obesity, hypertriglyceridemia, low serum high density lipoprotein cholesterol, elevated blood pressure, and elevated fasting plasma glucose [1]. The metabolic syndrome can cause or intensify cardiovascular, as well as renal disease [1,2]. One of the possible etiologies of the metabolic syndrome is insulin resistance associated with hyperinsulinemia [1-4]. The stimulatory effect of insulin on sympathetic drive [5], vascular smooth muscle growth [6] and sodium and water retention [7] and its inhibitory effect on prostacyclin synthesis [8] have been suggested to be involved in the pathogenesis of obesity-related hypertension.

Dopamine, a well recognized neurotransmitter in the central nervous system, is also an important modulator of renal and adrenal function, sodium balance, and blood pressure. Dopamine receptors are classified into two subfamilies: the D_1_-like receptor subfamily includes the D_1_ and the D_5_ receptors, while the D_2_, D_3_ and D_4_ receptors belong to the D_2_-like receptor subfamily. Dysfunction of the renal dopaminergic system is implicated in the pathogenesis and/or maintenance of hypertension [9,10].

The obese Zucker rat is a model of metabolic syndrome characterized by hyperinsulinemia, hyperglycemia, and hypertension. Dopamine has been implicated in the development of obesity, caused, in part, by increased food intake due to decreased dopaminergic function, specifically the D_2_ receptor, in the central nervous system [11,12]. D_1_ receptor mediated-natriuresis and diuresis are also impaired in obese Zucker rats [13,14]. Although the kidney is important in the long-term regulation of blood pressure [15], hypertension is also accompanied by increased vascular resistance [16]. Dopamine receptors are expressed in resistance vessels. In the rat mesenteric artery, D_1_ and D_5_ receptors are expressed in the tunica media while D_2_-like receptors are expressed mainly at the adventitia-tunica media transitional zone. The increase in blood pressure with aging has been related to a decrease in the expression of arterial D_1_, D_2_, and D_5_ receptor [17]. However, the role of the arterial D_1_ receptor in the hypertension of obese Zucker rats is not clear. Our current study tested the hypothesis that the vasorelaxant effect of the D_1_ receptor is impaired in obese Zucker rats.

## Methods

### Animal experiments

Male obese Zucker rats and age-matched male lean Zucker rats were housed in plastic cages and fed normal rodent chow and tap water. Twelve obese Zucker rats (six to eight-weeks old) were randomly divided into two groups: 1) one group consisted of obese rats treated (6 weeks) with oral rosiglitazone maleate (10 mg/kg, suspended in 1% carboxy methyl cellulose in distilled water); and 2) the other group consisted of obese control rats treated with the vehicle (1% carboxy methyl cellulose). Eighteen lean Zucker rats (eight to ten-weeks old) were randomly divided into three groups: 1) control rats fed normal diet and treated with vehicle; 2) rats fed control diet and treated with STZ (single intraperitoneal injection, 65 mg/kg); and 3) rats fed high-fat diet (with 50% fat-derived calories) for one month.

All experiments were approved by the Daping Hospital Animal Use and Care Committee and all procedures were approved by the Experimental Animals Committee of Daping Hospital.

### Blood pressure and plasma glucose and insulin measurements

After an overnight fast, obese and lean Zucker rats were anesthetized with pentobarbital (Nembutal) with an initial dose of 60 mg/kg, followed by constant infusion at 40 mg/kg/hr [18]. Following a tracheotomy, the left carotid artery was catheterized with polyethylene-50 tubing for blood pressure monitoring. Blood pressure and heart rate were allowed to stabilize for 10 minutes [19] before accepting the blood pressure and heart rate observed as baseline. The abdomen was opened to expose the abdominal aorta and blood samples were collected from the aorta in EDTA-coated tubes for measurement of plasma glucose and insulin, and then the rats were sacrificed by an overdose of pentobarbital (100 mg/kg body wt). Plasma glucose concentrations were determined by using Accu-Chek Advantage glucose monitoring system. Plasma insulin levels were measured by a rat insulin 96-well plate assay (Millipore Co., St Charles, MO).

### Preparation and study of small resistance arteries

Each rat was anesthetized with sodium pentobarbital (60 mg/kg). The entire mesenteric bed was removed carefully and placed in ice-cold physiological salt solution (PSS) containing (mM):NaCl 119, KCl 4.7, CaCl_2_·H_2_O 2.5, MgSO4·H_2_O 1.17, NaH_2_CO_3_ 25, KH_2_PO_4_ 1.18, EDTA 0.027, and glucose 5.5, adjusted to pH 7.4. The mesenteric artery was carefully and quickly dissected from the surrounding fat and connective tissues. Third-order branches of the superior mesenteric artery (resting arterial diameter: 200 ± 20 μm) were cut into rings approximately 2 mm in length, and mounted on 40 μm stainless-steel wires in an isometric Mulvany-Halpern small-vessel myograph (model 91 M610, J.P. Trading, Science Park, Aarhus, Denmark) [20]. One wire was attached to a force transducer and the other to a micrometer so that wall tension can be measured at a predetermined internal diameter. The rings were maintained in PSS at 37°C and continuously bubbled with oxygen (95%) and carbon dioxide (5%) (Carbogen) [21]. The dissecting procedures were performed with extreme care to protect the endothelium from inadvertent damage, proved by a normal response to acetylcholine (Ach) (10^-5^ Ach-induced relaxation >80% of basal values of arteries preconstricted with phenylephrine HCl [PHE, 10^-5^M]). In some vessels, the endothelium was removed by pulling a hair along the inside of the vessel; successful denudation of the endothelium was confirmed by the absence of relaxation with Ach (10^-5^M) [22]. Following mounting, the arterial ring was equilibrated in PSS for 1 hr at 37°C at a wall tension of 0.1 mN/mm. Based on preliminary data from >100 vessels, we confirmed that a normalized circumference (L_0_) = 0.9 L100 resulted in maximal active force development. The vessels were studied at L_0_ in all subsequent protocols. After determining the response to Ach, as indicated above, the vessels were rinsed three times with fresh PSS and allowed to recover to baseline for 15 minutes. In the first set of experiments, the rings were contracted with phenylephrine HCl (10^-5^M) and high-potassium PSS (KPSS, 125 mM) to obtain the maximal response. After obtaining the maximal response to PHE (10^-5^M), the response curves to fenoldopam, a D_1_ receptor agonist [9,10], were measured by a cumulative concentration-dependent protocol (10^-9^ to 10^-5^M). Response to every single concentration of fenoldopam was observed for 2 minutes. To test the vasorelaxant specific effects of D_1_ receptors, the arteries were incubated with the D_1_-like receptor antagonist SCH23390 (10^-7^M) for 30 minutes before fenoldopam treatment. In order to determine specificity of the vasodilatory effect of D_1_ receptor stimulation, we studied the vasodilation induced by sodium nitroprusside (SNP, 10^-10^ to 10^-4^M) in PHE-preconstricted mesenteric arteries.

### Cell and sample preparation

Embryonic thoracic aortic smooth muscle cells from normotensive Berlin–Druckrey IX rats (A10, ATCC, Hercules, CA) were cultured at 37°C in 95% air/5% CO_2_ atmosphere in Dulbecco’s Modified Eagle’s Medium. A10 cells (80% confluence) and mesenteric arteries from Zucker rats were flash frozen by liquid nitrogen and homogenized in ice-cold lysis buffer (5 ml/gm tissue) (20 mM Tris-HCl, pH 7.4; 2 mM EDTA, pH 8.0; 2 mM EGTA; 100 mM NaCl; 10 μg/ml leupeptin; 10 μg/ml aprotinin; 2 mM phenylmethylsulfonyl fluoride; 1% NP-40), sonicated, kept on ice for 1 hr, and centrifuged at 16,000 g for 30 minutes. All samples were stored at -70°C until use.

### Immunoblotting

After boiling the homogenates in sample buffer (35 mmol/L Tris-HCl, pH 6.8, 4% SDS, 9.3% dithiothreitol, 0.01% bromophenol blue, 30% glycerol) at 95°C for 5 min, 100 μg of protein were separated by SDS-PAGE (10% polyacrylamide), and then electroblotted onto nitrocellulose membranes (Bio-Rad). The blots were blocked overnight with 5% nonfat dry milk in phosphate buffered saline with Tween 20 (PBST) (0.05% Tween 20 in 10 mmol/l phosphate buffered (isotonic) saline) at 4°C with constant shaking, then incubated with polyclonal rabbit anti-rat D_1_ receptor antibodies (1:400 dilution; Millipore) overnight in the cold-room at 4°C. The membranes were then further incubated with infrared- labeled secondary antibodies (donkey anti-rabbit IRDye 800, Li-Cor Biosciences, Lincoln, NE) added to bind to the primary antibody at room temperature for 1 hr. The membranes were washed three times with PBST. The bound complex was detected using the Odyssey Infrared Imaging System (Li-Cor Biosciences). The images were analyzed using the Odyssey Application Software to obtain the integrated intensities.

### Determination of basal D_1_ receptor phosphorylation by co-immunoprecipitation

Equal amounts of lysates (1.0 mg protein/ml supernatant from A10 cells or mesenteric artery) were incubated with polyclonal antiphosphoserine antibody (HKSP, New Territories, HK) (2.5 μg/ml) for 1 hr and protein-G agarose at 4°C for 12 hr. The immunoprecipitates were pelleted and washed four times with lysis buffer. The pellets were suspended in sample buffer, boiled for 10 min, and subjected to immunoblotting with polyclonal affinity-purified rabbit anti-rat D_1_ receptor antibody. In order to determine the specificity of the bands, normal rabbit IgG (negative control) and D_1_ receptor antibody (positive control) were used as the immunoprecipitants [23]. The bound complexes were detected using the Odyssey Infrared Imaging System (Li-Cor Biosciences). The images were analyzed using the Odyssey Application Software to obtain the integrated intensities.

### Materials

Fenoldopam, rosiglitazone maleate, STZ, mitogen-activated protein kinase (MAPK) inhibitor PD98059, Ach, SNP, PHE, and D_1_ receptor antagonist SCH23390 were obtained from Sigma-Aldrich (St. Louis, MO). Rabbit anti-rat D_1_ receptor polyclonal antibodies and rat insulin 96 well plate assay kit were obtained from Millipore Corporation (St Charles, MO); and anti-phosphoserine antibodies were obtained from Abcam Ltd (New Territories, Hong Kong, China).

### Statistical analysis

The data are expressed as mean ± SEM. Comparison within groups was made by repeated measures ANOVA (or paired *t-*test when only 2 groups were compared), and comparison among groups (or *t*-test when only 2 groups were compared) was made by factorial ANOVA with Holm-Sidak test. A value of *P <* 0.05 was considered significant.

## Results

### D_1_ receptor-mediated vasorelaxant effect is impaired in mesenteric arteries from obese Zucker rats

**Figure 1:**
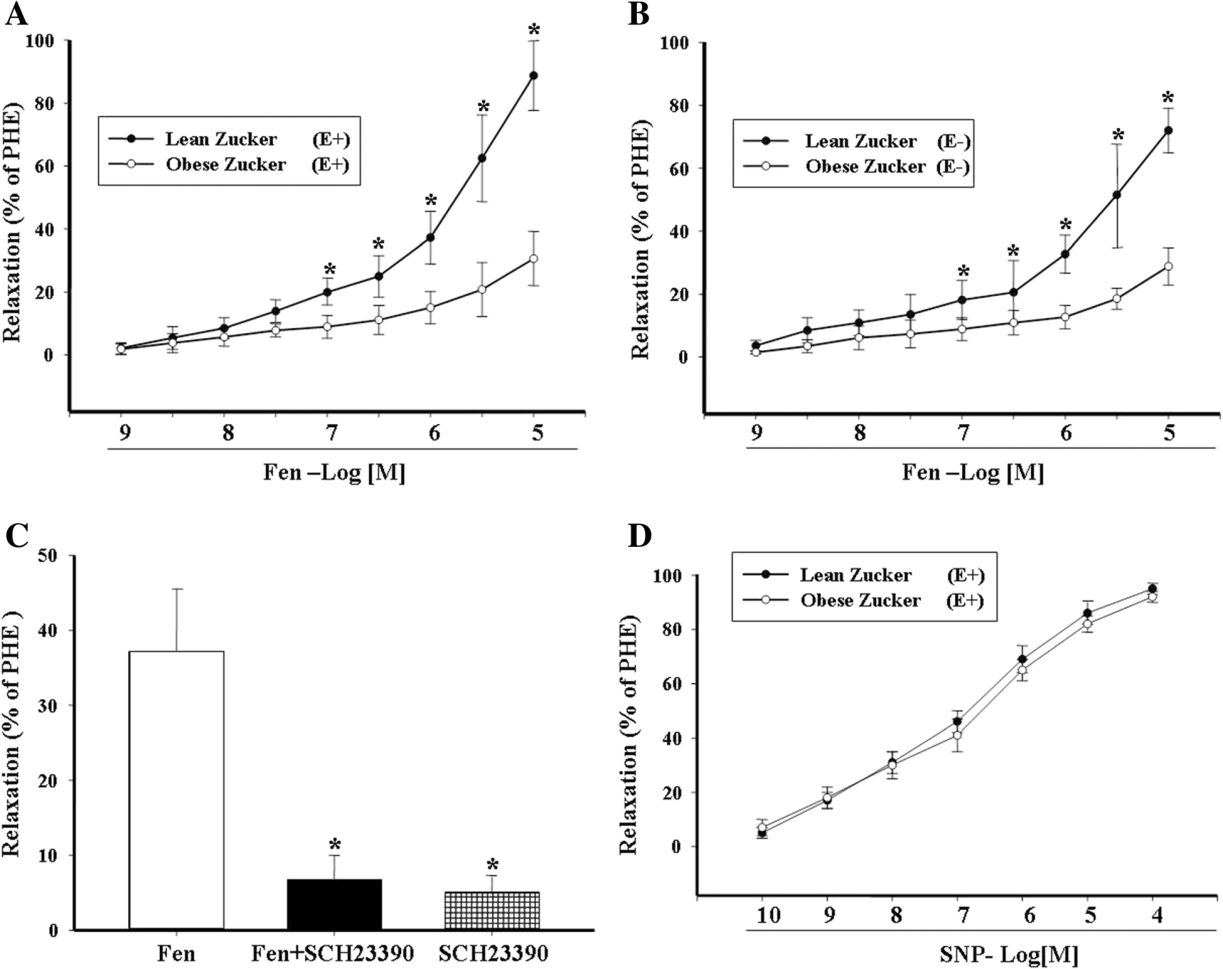
**Relaxation of phenylephrine (PHE)-preconstricted rat mesenteric arterial rings in response to fenoldopam and sodium nitroprusside (SNP).** Rat mesenteric arterial rings from obese and lean Zucker rats with (E+) **(A)** or without (E-) endothelium **(B)** were preconstricted with PHE (10^-5^M) and then treated with varying concentrations of fenoldopam (Fen, 10^-9^-10^-5^M) (*P < 0.01 vs. obese rats, n = 8). **(C):** D_1_-like receptor specificity of fenoldopam-induced vasorelaxation in rat mesenteric arterial rings. Rat mesenteric arterial rings from lean Zucker rats were preconstricted with PHE, and then incubated with fenoldopam (Fen, 10^-6^M) and/or SCH23390 (SCH, 10^-7^M) (*P < 0.01 vs. Fen, n = 6). **(D):** Rat mesenteric arterial rings from obese and lean Zucker rats with endothelium (E+) were preconstricted with PHE (10^-5^M) and then treated with varying concentrations of SNP (10^-10^-10^-4^M) (P = NS lean vs obese, n = 5).

Consistent with previous reports [24], several variables, including body weight, and fasting plasma glucose and insulin concentrations and blood pressure, were higher in obese than lean Zucker rats (Table 1). Fenoldopam, the D_1_ receptor agonist, (10^-7^M to 10^-5^M) induced a concentration-dependent vasorelaxation in the third-order mesenteric arteries from lean Zucker rats (Figure 1A). The absence of endothelium did not affect the fenoldopam-induced vasorelaxation, indicating that the fenoldopam-induced vasorelaxation was endothelium-independent (Figure 1B). The vasorelaxant effect of fenoldopam was via a D_1_-like receptor because a D_1_-like receptor antagonist, SCH23390 (10^-7^M), blocked the fenoldopam-induced vasorelaxation (Figure 1C), although SCH23390, by itself, was without any effect. The impaired vasorelaxant effect of fenoldopam was not a generalized phenomenon, because sodium nitroprusside (10^-10^ to 10^-4^M)-mediated vasodilation was not impaired in obese Zucker rats, consistent with previous reports [25,26] (Figure 1D).

**Table 1:** General and biochemical parameters of lean and obese Zucker rats (12-14 weeks old)

	**Lean control**	**Obese control**	**Obese+ROG**	**Lean+HFD**	**Lean+STZ**
Body weight (g)	273.83±5.37	439.64±7.34*	425.72±8.26	305.80±3.77*	222.79±3.38*
Plasma glucose (mmol/L)	5.86±0.17	8.88±0.48*	5.94±0.27^#^	6.22±0.15	>33.00*
Plasma insulin (ng/ml)	0.79±0.02	7.94±1.04*	2.97±0.44^#^	2.19±0.32*	0.46±0.06*
Mean blood pressure	118.91±1.85	138.26±3.62*	124.85±2.51^#^	120.59±2.39	129.66±1.51*

Values are means ±SE; n=6. *P < 0.05 vs. lean control rats; ^#^P < 0.05 vs. obese control rats.

Obese rats were treated with rosiglitazone (ROG): 10mg/kg/day for 6 weeks; Lean rats were treated with high fat diet (HFD) and streptozotocin (STZ) for 4 weeks.

**Figure 2:**
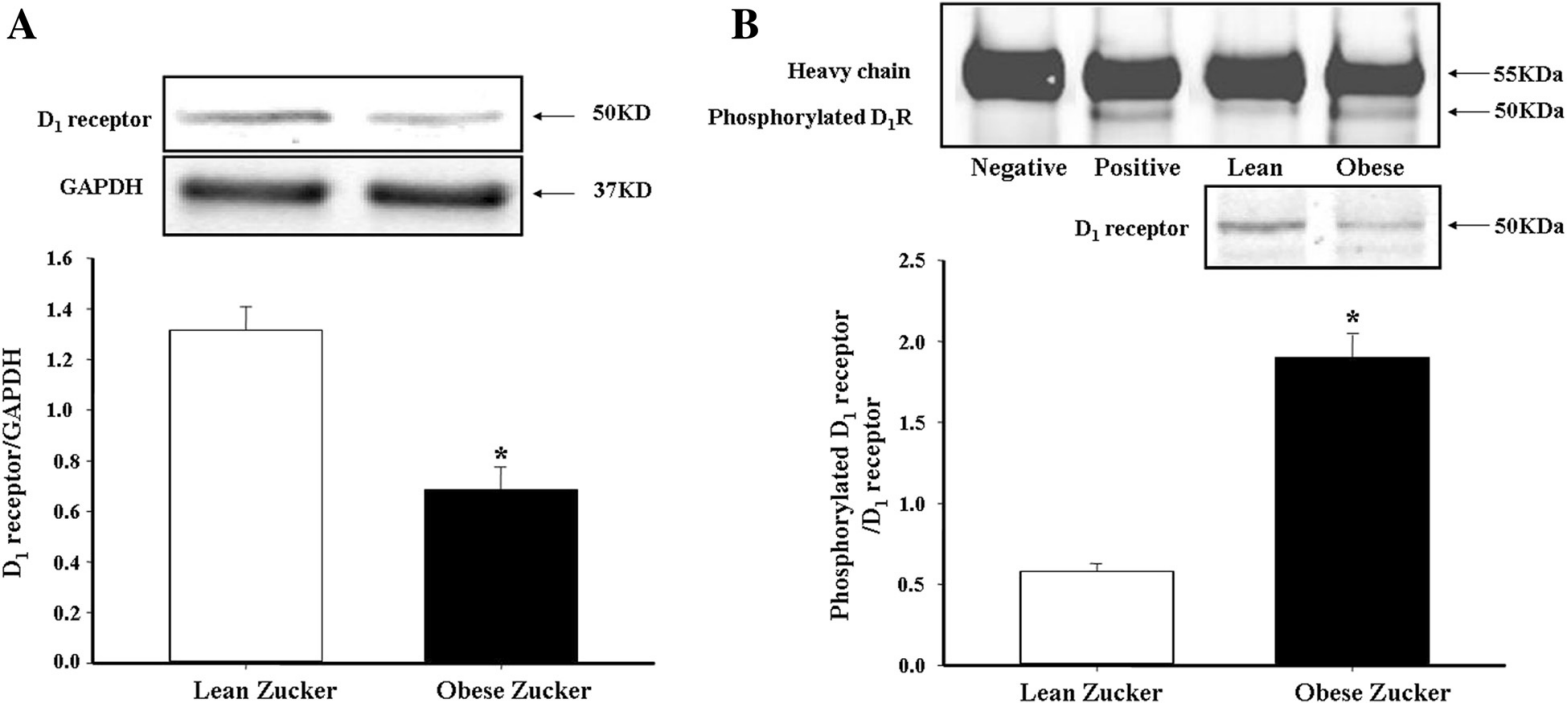
**D**_**1 **_**receptor expression and phosphorylation in mesenteric arteries from lean and obese Zucker rats. (A):** D_1_ receptor expression was determined by immunoblotting. **(B):** Serine-phosphorylated D_1_ receptor in rat mesenteric arteries was determined by co-immunoprecipitation and immunoblotting. Normal rabbit IgG (negative control) or D_1_ receptor antibody (positive control) was used as the immunoprecipitant. D_1_ receptor serine phosphorylation was normalized by D_1_ receptor protein. (*P < 0.05 vs. lean rats, n = 6).

To determine whether or not the arterial D_1_ receptor is involved in the pathogenesis of obesity-related hypertension, we studied the D_1_ receptor function in third-order mesenteric arteries from obese Zucker rats. We found that the fenoldopam-induced vasorelaxation was lost in obese Zucker rats in the presence (Figure 1A) or absence (Figure 1B) of the endothelium. The dysfunction of renal D_1_ receptor in hypertension [27] and obese Zucker rats [28,29] has been reported to be caused by basal hyperphosphorylation of the renal D_1_ receptor. To determine whether or not this phenomenon also exists in third order mesenteric arteries of obese Zucker rats, D_1_ receptor expression and phosphorylation was studied by immunoblotting and immunoprecipitation. We found that D_1_ receptor expression was lower (Figure 2A), while basal D_1_ receptor phosphorylation (Figure 2B) in third order mesenteric arteries was higher in obese than lean Zucker rats.

### Roles of hyperinsulinemia and hyperglycemia in the D_1_ receptor vascular dysfunction in obese Zucker rats

**Figure 3:**
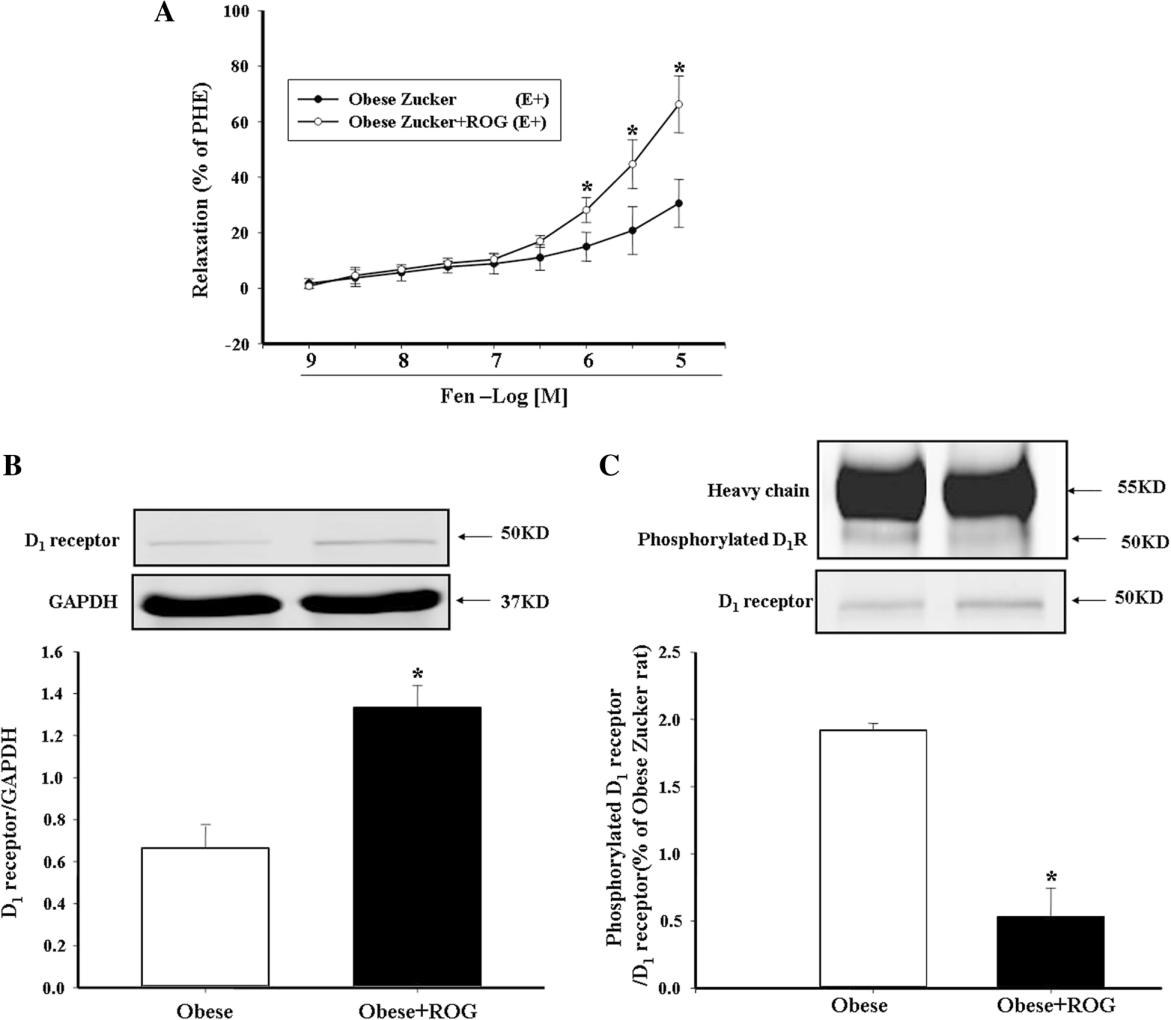
**Effect of rosiglitazone on D**_**1 **_**receptor expression and function in mesenteric arteries from obese Zucker rats.** The mesenteric arteries from obese Zucker rats, treated with rosiglitazone (ROG) or vehicle, were incubated with varying concentrations of fenoldopam (10^-9^-10^-5^M). The vasorelaxation with fenoldopam is shown in **3A** (*P < 0.05, vs. obese rats, n = 8 repeated measures ANOVA, Holm-Sidak test) while D_1_ receptor expression or phosphorylation in mesenteric arteries, determined by immunoblotting or co-immunoprecipitation, is shown in **3B and 3C,** respectively. D_1_ receptor serine phosphorylation was normalized by D_1_ receptor protein. (*P < 0.05, vs. obese rats, n = 6),

The pathogenesis of obesity is complex. To determine whether or not high plasma levels of glucose and insulin are involved in the obesity-related hypertension, the obese Zucker rats were treated with the insulin sensitizer [30], rosiglitazone (10 mg/kg oral, daily). We found that rosiglitazone restored the impaired fenoldopam-mediated vasorelaxation in obese Zucker rats (Figure 3A), increased the decreased D_1_ receptor expression (Figure 3B), and decreased the increased phosphorylation (Figure 3C) of D_1_ receptor in mesenteric arteries from obese Zucker rats consistent with those reported in the kidney [28].

**Figure 4:**
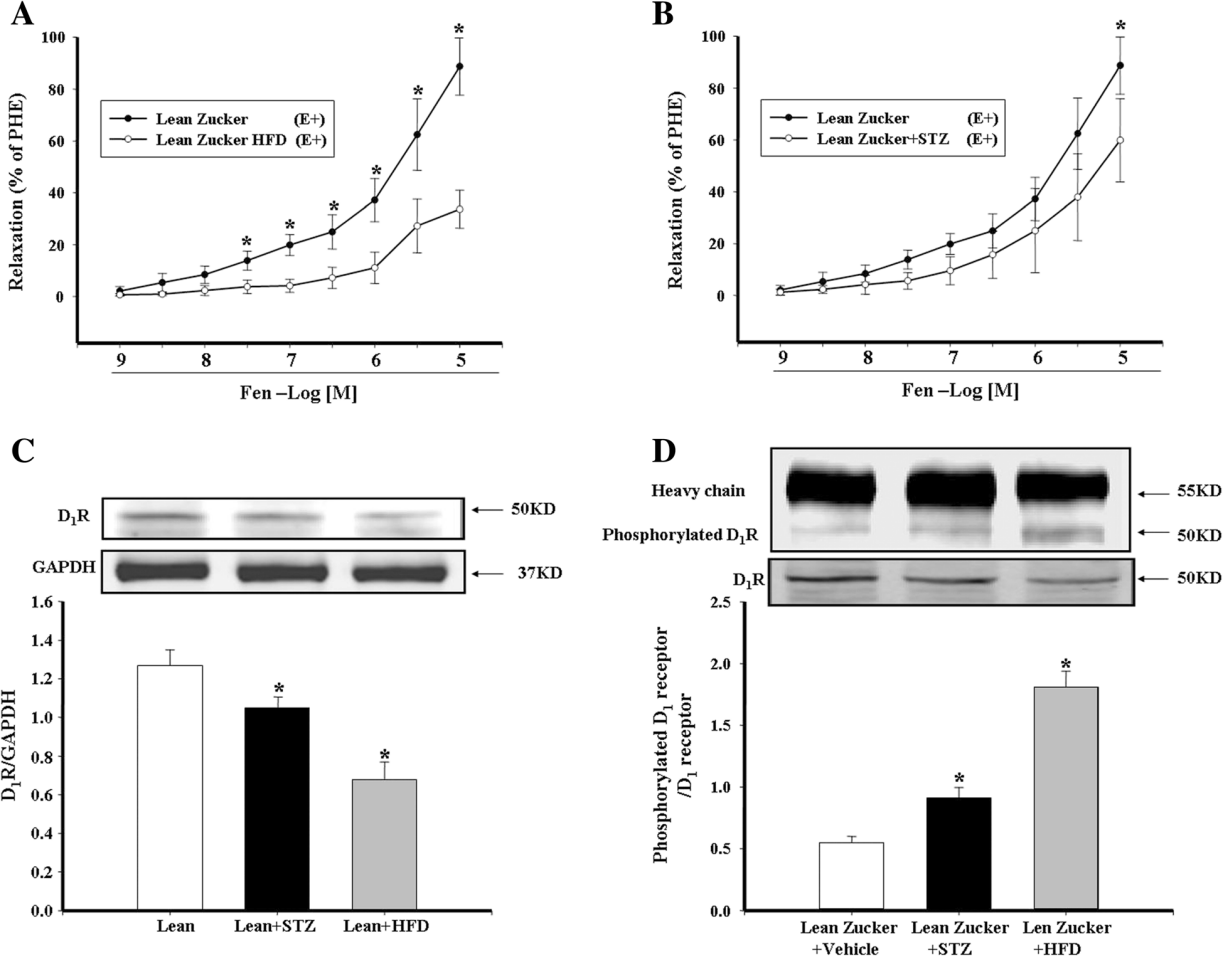
**D**_**1 **_**receptor expression and function in lean Zucker rats with hyperinsulinemia or hyperglycemia.** Lean Zucker rats were fed high-fat diet (HFD) to induce hyperinsulinemia or intraperitoneally injected with streptomycin (STZ) to induce hyperglycemia. The mesenteric arteries preconstricted with PHE from those rats were treated with varying concentrations of fenoldopam (Fen, 10^-9^-10^-5^M) **(A and B)** (*P < 0.05, vs. lean Zucker rats, n = 8). D_1_ receptor expression was determined by immunoblotting **(C)** and D_1_ receptor phosphorylation **(D)** was determined by co-immunoprecipitation and immunobloting. D_1_ receptor serine phosphorylation was normalized by D_1_ receptor protein (*P < 0.05, vs. obese rats, n = 6).

Consistent with other reports [28,30], our current study found that rosiglitazone reduced plasma insulin and glucose levels and blood pressure (Table 1). Therefore, it was difficult to determine whether hyperinsulinemia, hyperglycemia, or both led to the dysfunction of the arterial D_1_ receptor. To overcome this dilemma, we used the hyperinsulinemic and hyperglycemic lean Zucker rat models. Lean Zucker rats fattened by a high-fat diet for one month developed hyperinsulinemia but had normal blood pressure, similar to other reports [31,32]. To establish a hyperglycemia model, lean Zucker rats were intraperitoneally injected with a single dose of STZ (65 mg/kg) [33]; the STZ-treated lean Zucker rats would be expected to develop slightly high blood pressure and hyperglycemia but with low plasma insulin levels (Table 1), as described by others [34]. We found that both hyperinsulinemic (Figure 4A) and hyperglycemic (Figure 4B) lean Zucker rats had impaired D_1_ receptor-mediated vasorelaxation, accompanied by decreased D_1_ receptor expression (Figure 4C) and increased D_1_ receptor phosphorylation (Figure 4D), but the dysfunction of the D_1_ receptor was greater in the hyperinsulinemia than hyperglycemia model, indicating that insulin plays a more important role than hyperglycemia in the impairment of arterial D_1_ receptor-mediated relaxation (Figures 4A-D).

### Effect of insulin and glucose on D_1_ receptor expression and phosphorylation in A10 cells

**Figure 5:**
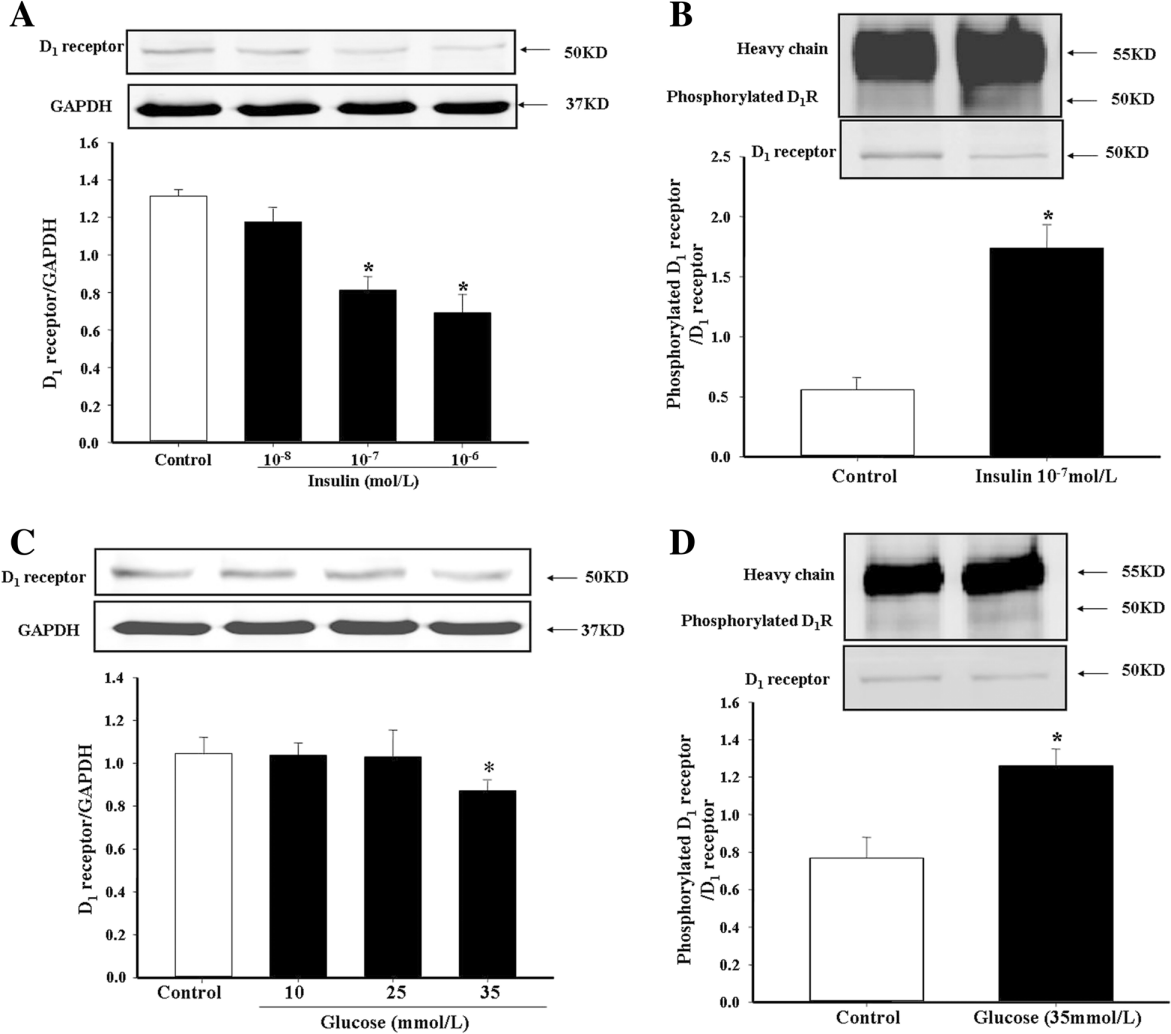
**Effect of insulin and glucose on D**_**1 **_**receptor expression and phosphorylation in A10 cells.** A10 cells were treated with varying concentrations of insulin **(A and B)** or glucose **(C and D)** for 24 hrs. D_1_ receptor expression was determined by immunoblotting. A10 cells were treated with insulin (10^-7^M) and glucose (35 mM) for 24 hrs. D_1_ receptor expression and phosphorylation were determined by co-immunoprecipitation or immunoblotting. D_1_ receptor serine phosphorylation was normalized by D_1_ receptor protein (*P < 0.05, vs. control, n = 5).

**Figure 6:**
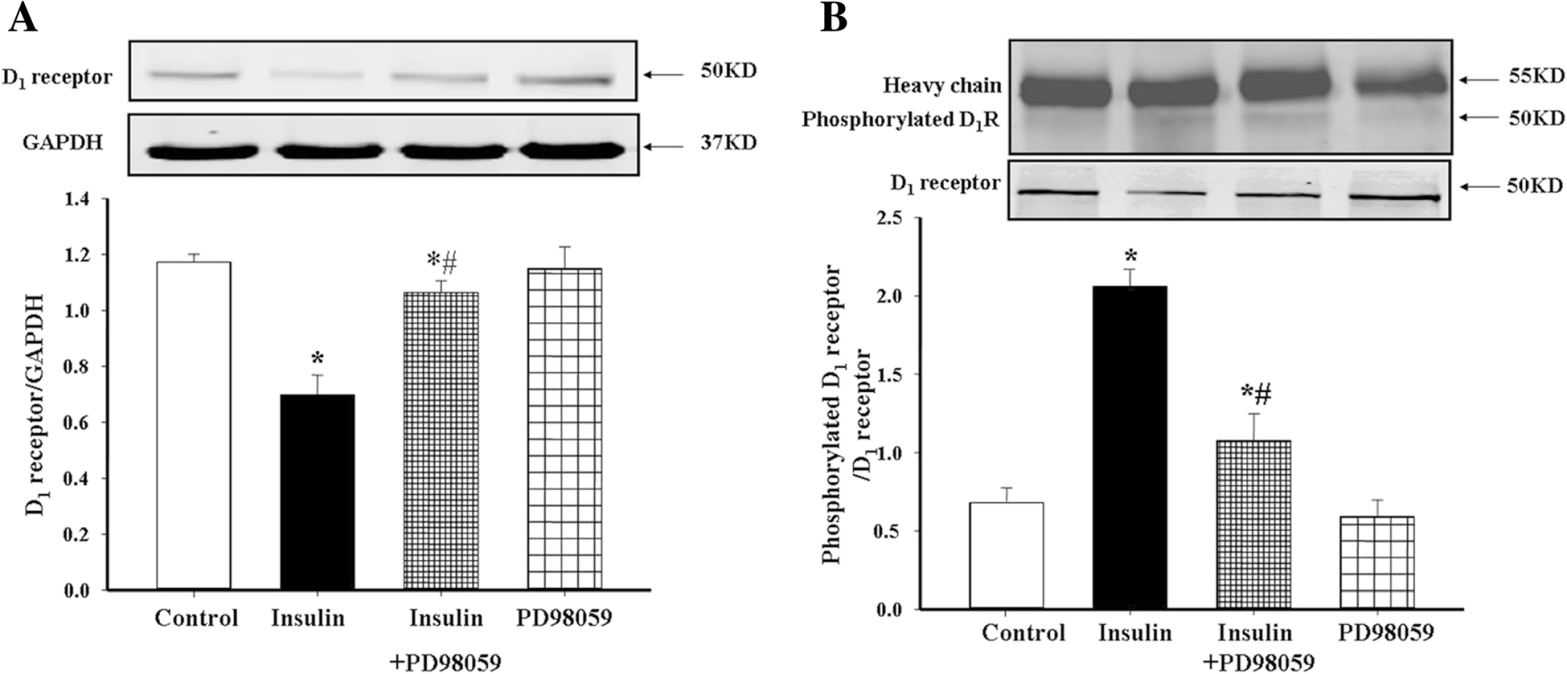
**Role of MAP kinase in the regulation by insulin of D**_**1 **_**receptor expression and phosphorylation in A10 cells.** A10 cells were treated with insulin (10^-7^M) in the presence or absence of the MAP kinase inhibitor PD98059 (10^-6^M) for 24 hrs. D_1_ receptor expression **(A)** or phosphorylation **(B)** was determined by immunoblotting or co-immunoprecipitation. D_1_ receptor serine phosphorylation was normalized by D_1_ receptor protein. (*P < 0.01, vs. control; ^#^P < 0.05, vs. insulin, n = 5).

To further confirm the *in vivo* results, the effects of high insulin and high glucose concentrations on D_1_ receptor expression and phosphorylation were studied in A10 cells, a rat thoracic aorta-derived smooth muscle cell line. Treatment with insulin (Figures 5A and 5B) or high glucose (Figures 5C and 5D) decreased D_1_ receptor expression and increased D_1_ receptor phosphorylation. In the presence of a MAP kinase inhibitor, PD98059 (10^-6^ M), the effects of insulin on D_1_ receptor expression and phosphorylation were blocked, indicating that MAP kinase is involved in the signaling pathway (Figures 6A and 6B).

## Discussion

### Role of hyperinsulinemia in obesity-related hypertension

Obesity is a well-known risk factor for hypertension [15,35]. Indeed, risk estimates according to the Framingham study show that roughly 80% of essential hypertension in men and 65% in women can be directly attributed to obesity [36]. It is accepted that insulin resistance is epidemiologically linked with hypertension [37,38]. The compensatory hyperinsulinemia that occurs with insulin resistance increases renal sodium reabsorption and sympathetic activity and leads to elevated arterial pressure [39]. Insulin resistance and compensatory hyperinsulinemia impair the production of nitric oxide and favor the production of vasoconstrictors [40-42]. Our current study found hyperinsulinemia, hyperglycemia, and elevated blood pressure in obese Zucker rats that were reduced by rosiglitazone treatment similar to previous reports [43,44]. We also found that lean Zucker rats fed a high fat diet had hyperinsulinemia and STZ-treated lean Zucker rats were hyperglycemic and hypoinsulinemic, similar to other reports [31,45].

### Impairment of D_1_ receptor-mediated vasodilation is involved in obesity-related hypertension

Dopamine has been reported as an important modulator of sodium balance, renal and adrenal function, and blood pressure and is relevant to the pathogenesis and/or maintenance of hypertension. In humans with essential hypertension and rodents with genetic hypertension (SHRs and Dahl salt-sensitive rats), D_1_-like receptor agonist-mediated natriuretic and diuretic responses are impaired [46,47]. The ability of D_1_-like receptors to stimulate adenylyl cyclase activity in renal arteries is also impaired in SHRs [48] and we have also reported an impaired ability of D_1_ receptors to dilate the mesenteric arteries of SHRs [49]. Other studies have shown that the D_1_ receptor is hyperphosphorylated and uncoupled from G protein subunits, leading to the D_1_ receptor dysfunction in SHRs, which precedes the onset of hypertension and co-segregates with the hypertensive phenotype [10,50]. Thus, D_1_ receptor dysfunction is a primary defect in the hypertension of SHRs. Lokhandwala *et al* have found that obese Zucker rats have defective renal D_1_ receptor function associated with a decrease in D_1_ receptor expression and ability to inhibit Na, K-ATPase and Na,H-exchanger activities in renal proximal tubules [13,28,30]. These authors suggested that the renal dopaminergic dysfunction in obese Zucker rats is acquired and not inherited [14] and could be ameliorated by inhibition of reactive oxygen species production by tempol and insulin sensitization by rosiglitazone [28,29]. Because increased peripheral vascular resistance and altered vascular reactivity are distinctive features of essential hypertension [16], we tested the hypothesis that the hypertension of obese Zucker rats may be caused by an impairment of D_1_ receptor-mediated vasodilation. This was indeed the case as the D_1_ receptor-mediated vasorelaxation was impaired in obese Zucker rats. This dysfunction of the D_1_ receptor could be ascribed to the decreased D_1_ receptor expression and increased D_1_ receptor phosphorylation because the rosiglitazone-mediated amelioration of the impaired D_1_ receptor relaxant effect was associated with an increase in D_1_ receptor expression and a decrease in D_1_ receptor phosphorylation. Rosiglitazone, a PPARγ agonist, has many effects on the cardiovascular system independent of its insulin sensitizing effects, including anti-inflammation and direct stimulation of NO production [51]. Whether or not such mechanisms were involved in the rosiglitazone-mediated improvement of the vasodilatory effect of the D_1_ receptor in the current report needs to be determined in a future study.

### Role of hyperinsulinemia or hyperglycemia in the dysfunction of arterial D_1_ receptor in obese Zucker rats

Obese Zucker rats have increased plasma insulin and fasting glucose levels [24,52,53]. As stated above, treatment of obese Zucker rats with rosiglitazone reduced plasma insulin and glucose levels and improved the vasorelaxant effect of fenoldopam that was associated with an increase in the vascular expression of D_1_ receptor and a decrease in vascular serine-phosphorylated D_1_ receptor. Thus, hyperinsulinemia and hyperglycemia may be involved in the defective fenoldopam-mediated relaxation in small mesenteric arteries isolated from obese Zucker rats. In order to identify the mechanisms that caused the impaired D_1_ receptor vasodilatory function in obese Zucker rats, we used two rat models: high fat diet-induced hyperinsulinemia and STZ-induced type 1 diabetes in lean Zucker rats. The former rat model is characterized by insulin resistance and hyperinsulinemia [31,32] while the latter rat model is characterized by hyperglycemia and low plasma insulin levels [33,34]. After the establishment of hyperinsulinemia or hyperglycemia, mesenteric arteries had an impaired fenoldopam-mediated vasorelaxation that was more evident in the high fat diet-fed hyperinsulinemic rats than STZ-induced type I diabetic rats. Therefore, both hyperinsulinemia and hyperglycemia are involved in the dysfunction of the arterial D_1_ receptor, but hyperinsulinemia plays a more important role than hyperglycemia in this phenomenon.

The ability of increased insulin and glucose concentrations to decrease D_1_ receptor expression and increase D_1_ receptor phosphorylation *in vivo* was confirmed *in vitro* in A10 cells. Treatment of A10 cells with insulin decreased D_1_ receptor expression and increased D_1_ receptor phosphorylation, consistent with the findings in renal proximal tubule cells [54,55]. Mitogen-activated protein kinase (MAPK) is activated in cardiovascular diseases such as diabetes and hypertension [56,57]. The up-regulation of MAPK reduces renal D_1_ receptor affinity and G-protein coupling in obese rats [58]. The current studies also showed that blockade of MAPK reversed both the decreased D_1_ receptor expression and increased D_1_ receptor phosphorylation caused by high insulin in A10 cells.

### Limitations

Besides of the role of hyperinsulinemia and hyperglycemia in the D_1_-receptor mediated arterial dysfunction, adiponectin, as an important adipose tissue-derived factor, might have some effect on dopamine function. For example, monosodium glutamate induces obesity in rodents markedly decreases adiponectin levels [59] and compromises dopaminergic systems [60]. Moreover, dopamine stimulates adiponectin release [61], and antidiabetic treatment in Zucker diabetic fatty rats inhibits the development of hypo-adiponectinemia in mesenteric resistance arteries, but is not able to improve adiponectin induced vasodilation [62]. Whether or not the plasma adiponectin would affect arterial dopamine receptor function needs to be determined in the future study.

## Conclusions

In summary, this study shows that both hyperinsulinemia and hyperglycemia impair vascular D_1_ receptor function that is associated with decreased D_1_ receptor expression and increased D_1_ receptor phosphorylation. Besides, hyperinsulinemia plays a more important role than hyperglycemia in the dysfunction of the arterial D_1_ receptor in obese Zucker rats. Impaired D_1_ receptor-mediated vasorelaxation is involved in the pathogenesis of obesity-related hypertension.

## Abbreviations

STZ: Streptomycin; HFD: High-fat diet; MS: Metabolic syndrome; PSS: Physiological salt solution; Ach: Acetylcholine; PHE: Phenylephrine HCl; KPSS: High-potassium PSS; SNP: Sodium nitroprusside; MAPK: Mitogen-activated protein kinase; ROG: Rosiglitazone; Fen: Fenoldopam; SCH: SCH23390; E: Endothelium; M: mol/L

## Competing interests

The authors declare that they have no competing interests.

## Authors’ contributions

JJF, YH, HYW performed most of the experiments and analyzed data and wrote the manuscript. ZW performed some experiments and write the cover letter. YKL reviewed and edited the manuscript. XJC and YC performed some experiments and contributed to the discussion. Laureano D. Asico and Pedro A. Jose edited the manuscript and contributed to the discussion. CCZ and LZ designed the experiments and wrote and edited the manuscript. All authors read and approved the final manuscript.
